# The Consumption of Bicarbonate-Rich Mineral Water Improves Glycemic Control

**DOI:** 10.1155/2015/824395

**Published:** 2015-12-21

**Authors:** Shinnosuke Murakami, Yasuaki Goto, Kyo Ito, Shinya Hayasaka, Shigeo Kurihara, Tomoyoshi Soga, Masaru Tomita, Shinji Fukuda

**Affiliations:** ^1^Systems Biology Program, Graduate School of Media and Governance, Keio University, 5322 Endo, Fujisawa, Kanagawa 252-0882, Japan; ^2^Institute for Advanced Biosciences, Keio University, 246-2 Mizukami, Kakuganji, Tsuruoka, Yamagata 997-0052, Japan; ^3^Onsen Medical Science Research Center, Japan Health and Research Institute, 1-29-4 Kakigaracho, Nihonbashi, Chuo-ku, Tokyo 103-0014, Japan; ^4^Ito Medical Office, 7985-5, Nagayu, Naoirimachi, Taketa, Oita 878-0402, Japan; ^5^Faculty of Human Life Sciences, Tokyo City University, 8-9-18 Todoroki, Setagaya-ku, Tokyo 158-8586, Japan

## Abstract

Hot spring water and natural mineral water have been therapeutically used to prevent or improve various diseases. Specifically, consumption of bicarbonate-rich mineral water (BMW) has been reported to prevent or improve type 2 diabetes (T2D) in humans. However, the molecular mechanisms of the beneficial effects behind mineral water consumption remain unclear. To elucidate the molecular level effects of BMW consumption on glycemic control, blood metabolome analysis and fecal microbiome analysis were applied to the BMW consumption test. During the study, 19 healthy volunteers drank 500 mL of commercially available tap water (TW) or BMW daily. TW consumption periods and BMW consumption periods lasted for a week each and this cycle was repeated twice. Biochemical tests indicated that serum glycoalbumin levels, one of the indexes of glycemic controls, decreased significantly after BMW consumption. Metabolome analysis of blood samples revealed that 19 metabolites including glycolysis-related metabolites and 3 amino acids were significantly different between TW and BMW consumption periods. Additionally, microbiome analysis demonstrated that composition of lean-inducible bacteria was increased after BMW consumption. Our results suggested that consumption of BMW has the possible potential to prevent and/or improve T2D through the alterations of host metabolism and gut microbiota composition.

## 1. Introduction

Self-medication is an important approach to maintain and promote human health. There are many strategies of self-medication such as improving one's lifestyle and/or dietary habits, consumption of functional foods/beverages, and getting adequate exercise [1, 2]. Hot spring water and natural mineral water are traditionally used in public baths and balneotherapy in many countries. Balneotherapy is the use of thermal and/or mineral water derived from natural springs or drilled wells for treatment of human health by employing various methods such as bathing, drinking, mud therapy, and inhalation [3]. According to previous studies, it has been reported that balneotherapy has beneficial effects for various diseases such as type 2 diabetes (T2D) [4–6], rheumatism [7], low back pain [3], and cardiovascular disease [8, 9]. Particularly, consumption of bicarbonate-rich mineral water (BMW) has been reported to prevent or improve T2D [4, 6]. However, as the reported benefits of BMW were determined epidemiologically and clinically, the molecular mechanisms of the beneficial effects behind BMW remain unclear.

Metabolomics is a method of comprehensive measurement of metabolites and it has been known to be a powerful tool to gain new insights into various research fields such as metabolic syndrome [10], cancer [11, 12], chronic kidney disease [13], and biomarker screening [14]. A recent study has reported that plasma metabolome profiles were altered between healthy people and T2D patients [15]. Moreover, it has been also reported that gut microbial composition and function in T2D patients are different from that of healthy subjects [16–18]. Therefore, preventive and/or therapeutic effects for T2D derived from BMW consumption are expected via influencing blood metabolites concentrations and gut microbial compositions.

For this reason, blood metabolome analysis and gut microbiome analysis were included in our study to elucidate the molecular level effects of BMW consumption on glycemic control. In this study, BMW consumption test was conducted amongst 19 healthy volunteers. Here we show that serum glycoalbumin levels were decreased and 19 metabolites including glycolysis-related metabolites and 3 amino acids were changed after consumption of BMW. Additionally, microbiome analysis demonstrated that composition of lean-inducible bacteria, family Christensenellaceae, was increased after BMW consumption. This current study is an important study reporting the molecular level effects derived from consumption of BMW.

## 2. Materials and Methods

### 2.1. Sample Preparation

This study was approved by the Ethical Committees of Japan Health and Research Institute and Keio University Shonan Fujisawa Campus. All subjects were informed of the purpose of this study, and written consent was obtained from all subjects.

In this study, BMW consumption test was conducted amongst 19 healthy subjects (7 men and 12 women, ages from 26 to 59, 47 years old on the average). Initially, individual identification numbers were randomly assigned to 26 volunteers from N01 to N26; however N12 and N25 dropped out due to personal reasons. Additionally, N01, N02, N14, N18, and N21 were excluded from analysis because they forgot to drink TW and/or BMW at least once. BMW was obtained from Nagayu hot spring (Taketa, Oita, Japan) as it contains one of the highest bicarbonate concentrations in Japan. TW was purchased from Nishikawa water treatment plant (Yamagata, Japan). Mineral contents and pH of the TW and BMW used in this study were measured by the Hot Spring Research Center (Tokyo, Japan) and are as shown in Table 1. BMW were collected in 500 mL plastic bottles and stored in refrigerator until consumption. All BMW were consumed within 10 days from bottling. TW bottles were also similarly stored in refrigerator until consumption. During the test, volunteers opened 1 plastic bottle of TW or BMW every day and drank the 500 mL of TW or BMW divided into thrice daily (30–60 minutes before breakfast, lunch, and dinner). TW consumption periods and BMW consumption periods lasted for a week each and this cycle was repeated twice. Volunteers were instructed to keep to their normal dietary habits during the test, but consumption of medicinal drugs was prohibited.

Blood and fecal samples were collected on the first day of the test and last days of every week. Blood samplings that were collected from the same subject were performed at approximately the same time during the test. Body weight, body mass index (BMI), height, abdominal circumference, and blood pressure were measured on the first day of the test (see Table S1 of the Supplementary Material available online at http://dx.doi.org/10.1155/2015/824395). A schematic representation of the experimental design is as shown in Figure S1 of Supplementary Material.

### 2.2. Clinical Blood Tests

Clinical blood tests were performed at each sampling point, including measurement of fasting plasma glucose, serum glucose, glycoalbumin, insulin, total cholesterol, HDL cholesterol, LDL cholesterol, triglyceride, urate, sodium, chlorine, calcium, magnesium, and cortisol. The measurement of the concentrations of these parameters was outsourced to RINTEC Co., Ltd. (Fukuoka, Japan).

Plasma glucose measurement was performed using morning fasting blood samples obtained from only 8 volunteers. For these samples, the homeostasis model assessment ratio (HOMA-R) was calculated from plasma glucose and insulin levels.

### 2.3. Metabolome Analysis

Metabolome analysis was conducted as described previously with some modifications [13]. In brief, to extract metabolites from blood, 400 *μ*L of methanol including the internal standards (20 *μ*M each of methionine sulfone and D-camphor-10-sulfonic acid (CSA)) was added to the 40 *μ*L of blood samples. Next, this mixture was then mixed with 120 *μ*L of ultrapure water and 400 *μ*L of chloroform before centrifuging at 10,000 ×g for 3 min at 4°C. Subsequently, the aqueous layer was transferred to a centrifugal filter tube (UltrafreeMC-PLHCC 250/pk for Metabolome Analysis, Human Metabolome Technologies) to remove protein and lipid molecules. The filtrate was centrifugally concentrated and dissolved in 20 *μ*L of ultrapure water that contained reference compounds (200 *μ*M each of 3-aminopyrrolidine and trimesic acid) immediately before capillary electrophoresis with electrospray ionization time-of-flight mass spectrometry (CE-TOFMS) analysis.

The measurement of extracted metabolites in both positive and negative modes was performed by CE-TOFMS. All CE-TOFMS experiments were performed using the Agilent CE capillary electrophoresis system (Agilent Technologies). Annotation tables were produced from measurement of standard compounds and were aligned with the datasets according to similar *m*/*z* value and normalized migration time. Then, peak areas were normalized against those of the internal standards methionine sulfone or CSA for cationic and anionic metabolites, respectively. Concentrations of each metabolite were calculated based on their relative peak areas and concentrations of standard compounds.

After statistical analysis, metabolite set enrichment analysis (MSEA) [19] was performed using the metabolites that were significantly different between TW and BMW consumption periods.

### 2.4. DNA Isolation

Fecal DNA isolation was performed as described previously with some modifications [20]. Briefly, fecal samples were initially lyophilized by using VD-800R lyophilizer (TAITEC) for at least 18 hours. Freeze-dried feces were disrupted with 3.0 mm Zirconia Beads by vigorous shaking (1,500 r.p.m. for 10 min) using Shake Master (Biomedical Science). Fecal samples (10 mg) were suspended with DNA extraction buffer containing 200 *μ*L of 10% (w/v) SDS/TE (10 mM Tris-HCl, 1 mM EDTA, and pH 8.0) solution, 400 *μ*L of phenol/chloroform/isoamyl alcohol (25 : 24 : 1), and 200 *μ*L of 3 M sodium acetate. Feces in mixture buffer were further disrupted with 0.1 mm zirconia/silica beads by vigorous shaking (1,500 r.p.m. for 5 min) using Shake Master. After centrifugation at 17,800 ×g for 5 min at room temperature, bacterial genomic DNA was purified by the standard phenol/chloroform/isoamyl alcohol protocol. RNAs were removed from the sample by RNase A treatment and then DNA samples were purified once more by the standard phenol/chloroform/isoamyl alcohol protocol.

### 2.5. 16S rRNA Gene Sequencing

16S rRNA genes in the fecal DNA samples were analyzed using the MiSeq sequencer (Illumina). The V1-V2 region of the 16S rRNA genes was amplified from the DNA isolated from feces using bacterial universal primer set 27Fmod (5′-AGRGTTTGATYMTGGCTCAG-3′) and 338R (5′-TGC­TGC­CTC­CCG­TAG­GAG­T-3′) [21]. PCR was performed with Tks Gflex DNA Polymerase (Takara Bio Inc.) and amplification proceeded with one denaturation step at 98°C for 1 min, followed by 20 cycles of 98°C for 10 s, 55°C for 15 s, and 68°C for 30 s, with a final extension step at 68°C for 3 min. The amplified products were purified using Agencourt AMPure XP (Beckman Coulter) and then further amplified using forward primer (5′-AAT­GAT­ACG­GCG­ACC­ACC­GAG­ATC­TAC­AC-NNN­NNN­NN-TAT­GGT­AAT­TGT-AGR­GTT­TGA­TYM­TGG­CTC­AG-3′) containing the P5 sequence, a unique 8 bp barcode sequence for each sample (indicated in N), Rd1 SP sequence and 27Fmod primer and reverse primer (5′-CAA­GCA­GAA­GAC­GGC­ATA­CGA­GAT-NNN­NNN­NN-AGT­CAG­TCA­GCC-TGC­TGC­CTC­CCG­AGG­AGT-3′) containing the P7 sequence, a unique 8-bp barcode sequence for each sample (indicated in N), Rd2 SP sequence, and 338R primer. After purification using Agencourt AMPure XP, mixed sample was prepared by pooling approximately equal amounts of PCR amplicons from each sample. Finally, MiSeq sequencing was performed according to the manufacturer's instructions. In this study, 2 × 300 bp paired-end sequencing was employed.

### 2.6. Analysis of 16S rRNA Gene Sequences

Initially, to assemble the paired-end reads, fast length adjustment of short reads (FLASH) (v1.2.11) [22] was used. Assembled reads with an average *Q*-value < 25 were filtered out using in-house script. 5,000 filter-passed reads were randomly selected from each sample and used for further analysis. Reads were then processed using quantitative insights into microbial ecology (QIIME) (v1.8.0) pipeline [23]. Sequences were clustered into operational taxonomic units (OTUs) using 97% sequence similarity and OTUs were assigned to taxonomy using RDP classifier.

### 2.7. Statistical Analysis

To analyze the intraindividual alterations, statistical evaluation between two groups was performed by Wilcoxon signed-rank test (nonparametric paired test) using the R package exactRankTests (available at https://cran.r-project.org/web/packages/exactRankTests/index.html) or XLSTAT (v2014.6.04) (Addinsoft). For multiple comparisons, the data were analyzed using Friedman's test and post hoc Nemenyi test using XLSTAT. All statements indicating significant differences show at least a 5% level of probability.

### 2.8. Nucleotide Sequence Accession Number

The microbiome analysis data have been deposited at the DDBJ Sequence Read Archive (http://trace.ddbj.nig.ac.jp/dra/) under accession number DRA004008.

## 3. Results

### 3.1. Serum Glycoalbumin Levels Were Decreased after BMW Consumption

**Figure 1:**
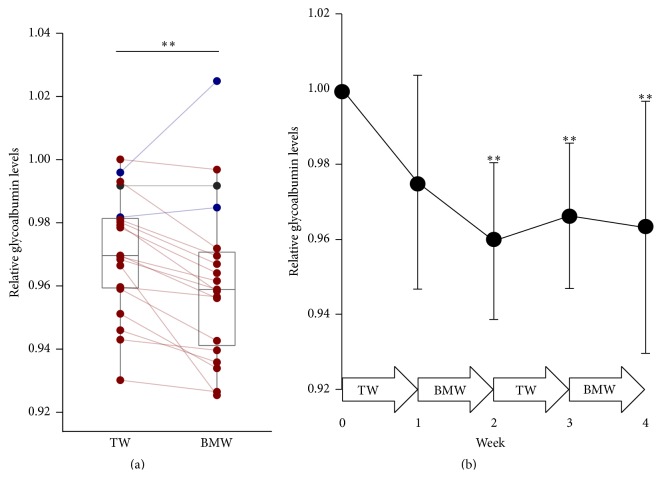
Comparisons of relative serum glycoalbumin levels between TW and BMW consumption periods. Glycoalbumin levels were expressed as a relative value to week 0. (a) Individual data of mean relative glycoalbumin levels of weeks 1 and 3 (TW) and weeks 2 and 4 (BMW) were shown in dot plots overlaid on box plots. Plots corresponding to the same individuals were connected with red, blue, or gray lines when the values were decreased, increased, or not changed in BMW consumption periods as compared with TW consumption periods, respectively. Plots were also colored in the same color as their lines. (b) Records of weekly glycoalbumin levels. Data were expressed as mean ± standard deviation (SD). Significances between week 0 and each week were shown at top of the graph. ^*∗∗*^ *P* < 0.005.

Firstly, intraindividual alterations of clinical parameters were analyzed. Serum glycoalbumin levels, one of the indexes of glycemic controls, were significantly decreased during BMW consumption periods as compared with TW consumption periods (Figure 1(a)). Serum glucose levels were not decreased significantly but tended to be lowered (*P* value = 0.092). Other parameters related to glycemic controls, like plasma glucose levels, insulin concentrations, and HOMA-R, were not different between TW and BMW consumption periods. In this study, other biochemical parameters involved with hypercholesterolemia, hyperuricemia, mineral consumption, and stress were also measured, but consumption of BMW did not have any impact on the above-mentioned parameters apart from blood calcium levels (Table 2). These results suggested that BMW consumption has the possible potential to prevent and/or improve T2D without changing insulin secretion and insulin resistance.

Although the serum glycoalbumin levels were significantly decreased during BMW consumption periods as compared with TW consumption periods, reduction of serum glycoalbumin levels was also observed after TW consumption periods as compared with before the test (week 0) (Figure 1(b)). Relative serum glycoalbumin levels decreased after the first TW consumption period (week 1) and further decreased after first BMW consumption period (week 2). Subsequently, it slightly increased after second TW consumption period (week 3) and then decreased again after second BMW consumption period (week 4). Taken together, these results suggest that the habit of water consumption before every meal has the possible potential to decrease serum glycoalbumin levels, but BMW consumption is more effective.

### 3.2. BMW Consumption-Related Changes of Physiological Metabolism

**Figure 2:**
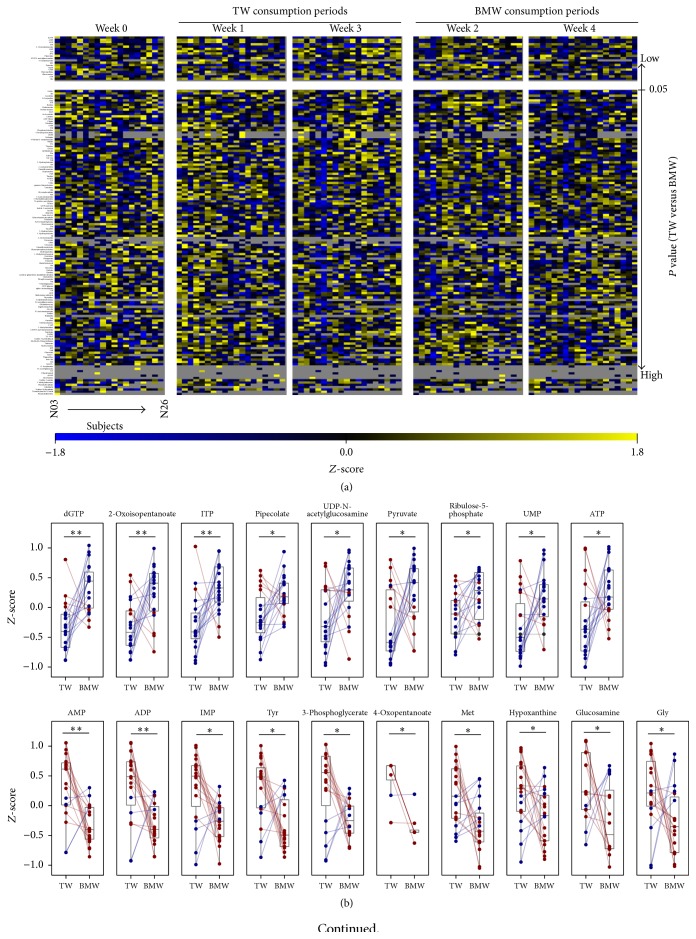
Comparisons of blood metabolites between TW and BMW consumption periods. (a) Relative concentrations of each metabolite in blood were transformed to *Z*-score by subjects and shown as heat maps using blue-black-yellow scheme. Gray color indicates that the metabolites were not detected in the sample. A total of 152 metabolites were arranged in increasing order of *P* value that was calculated by Wilcoxon signed-rank test between TW and BMW consumption periods. (b) Mean relative concentrations of each metabolite (*Z*-score) of weeks 1 and 3 (TW) and weeks 2 and 4 (BMW) were shown in dot plots overlaid on box plots in the same manner as in Figure 1(a). Only 19 metabolites that their concentrations were significantly increased (upper 9 metabolites) or decreased (lower 10 metabolites) after BMW consumption (shown in upper block of panel (a)) were demonstrated. (c) Relative concentrations of 3-hydroxybutyrate in blood were shown in dot plots overlaid on box plots in the same manner as in Figure 1(a). NS: not significant; ^*∗*^ *P* < 0.05; ^*∗∗*^ *P* < 0.005.

To evaluate the molecular level effects of BMW consumption, blood metabolome analysis was performed using CE-TOFMS. A total of 152 metabolites were detected from blood samples at least from 1 subject and 1 time point and concentrations of these metabolites were compared within subject. Over 85% of metabolites were not significantly changed after BMW consumption periods and it was expected that the concentrations of most metabolites remained consistent due to physiological homeostasis (Figure 2(a)). However, the concentrations of 19 metabolites were significantly different between TW and BMW consumption periods (Figures 2(a) and 2(b)). These effects were expected to be derived from BMW consumption. As compared with TW consumption periods, 9 metabolites were significantly increased and 10 metabolites were significantly decreased in BMW consumption periods (Figure 2(b)). Metabolites that may be related to T2D such as glycolysis-related metabolites (pyruvate, ATP, and ADP), amino acids (tyrosine, methionine, and glycine), and UDP-N-acetylglucosamine were included in significantly changed metabolites. In addition, 3 amino acids were significantly decreased, but almost all amino acids were also lowered after BMW consumption periods (see Figure S2 of Supplementary Material).

Additionally, MSEA was performed using the 19 significantly changed metabolites (Table 3). These metabolites were involved with central metabolic pathways (glycolysis, gluconeogenesis, and citric acid cycle) and nitrogen metabolism pathways such as ammonia recycling, urea cycle, and methionine metabolism. These results suggested that physiological metabolism was modified by consumption of BMW.

On the other hand, it was observed that 3-hydroxybutyrate, one of the indexes of diabetic ketoacidosis, was not altered by BMW consumption (Figure 2(c)).

### 3.3. BMW Consumption-Related Changes of Microbiota Compositions

**Figure 3:**
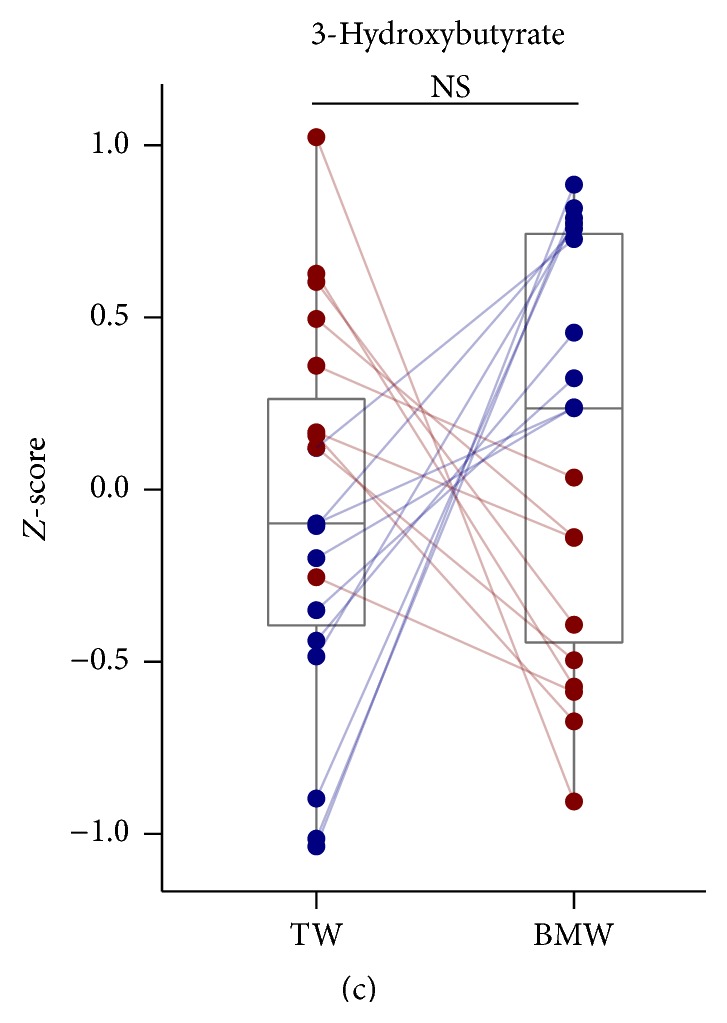
Comparisons of fecal microbiota compositions between TW and BMW consumption periods. (a) Family level compositions of fecal microbiota during the test. (b) Mean relative abundances of each taxa (*Z*-score) of weeks 1 and 3 (TW) and weeks 2 and 4 (BMW) were shown in dot plots overlaid on box plots in the same manner as in Figure 1(a). Only 8 families that their compositions were significantly different between TW and BMW consumption periods were demonstrated. ^*∗*^ *P* < 0.05; ^*∗∗*^ *P* < 0.005.

Microbiome analysis was conducted using fecal samples that were collected weekly during the study. A total of 7,075 OTUs were constructed from 16S rRNA gene sequences derived from 19 subjects. These OTUs corresponded to 62 families and whole structures of fecal microbiota are shown (Figure 3(a)). To investigate the intraindividual changes of gut microbiota during the test, relative abundances of each microbial taxon were compared between TW and BMW consumption periods within subjects. From the results, it was observed that over 85% of families were not altered similarly to blood metabolites, but relative proportions of 8 families especially Christensenellaceae were significantly different between TW and BMW consumption periods (Figure 3(b)). These results suggested that consumption of BMW has a potential to change some gut microbial compositions.

## 4. Discussion

In the present study, it was shown that serum glycoalbumin levels were significantly decreased after BMW consumption as compared with TW consumption. Although glycoalbumin levels have been known to reflect blood glucose levels during the last 14 days of the experiment [24], the mean blood glucose levels were not decreased significantly but tended to be lowered by BMW consumption as observed in our data. This might be attributed to the fact that blood glucose levels are influenced by external factors such as food intake and exercise [25]. Although insulin is one of the major factors involved in blood glucose control, the reduction of glycoalbumin level that was observed in this study was not expected to be related to the changing of insulin secretion and/or insulin resistance because insulin concentrations and HOMA-R were not significantly changed after BMW consumption. Previous studies have also reported the reduction of blood glucose levels by consumption of BMW or sulfate containing water [4, 5], but this study is the first evidence of reduction of serum glycoalbumin levels by consumption of BMW. Additionally, relative glycoalbumin levels were partly decreased even after TW consumption period. Although it has been previously reported that body weights of the volunteers were decreased by consumption of TW before every meal during 12 weeks [26], the reduction in serum glycoalbumin levels is a novel result.

Clinical tests also indicated significant increments in blood calcium levels. This phenomenon was expected to be attributed to BMW consumption because BMW used in this study contains calcium (177 mg/kg). According to the previous report, calcium deficiency may apparently lead to insulin resistance [27]. Therefore, calcium supplementation by drinking mineral water that includes calcium may be important as well as BMW consumption to better manage glycemic control.

According to the metabolome analysis of blood samples, ATP and pyruvate were significantly increased whereas ADP was decreased. This result suggested that glycolysis was upregulated after consumption of BMW. Since blood glucose levels were not decreased significantly but tended to be lowered as observed in our results, the effects of glycolysis enhancement were expected. On the other hand, concentrations of 3 amino acids (tyrosine, methionine, and glycine) were significantly lowered after consumption of BMW. A previous study reported that high concentrations of various amino acids especially tyrosine in blood are one of the risk factors of T2D [28]. Additionally, it has been also reported that plasma concentrations of several amino acids including tyrosine and methionine were significantly high in hyperinsulinemia (it is often observed in early stage of T2D) patients as compared to healthy subjects [29]. Our results demonstrated that concentrations of 3 amino acids including tyrosine and methionine in blood were significantly decreased and that those of other standard amino acids were tended to be lowered after consumption of BMW. As such, it can be suggested that the BMW consumption has a possible potential to prevent and/or improve T2D through the alterations of metabolism in the body.

After insulin resistance was increased, it can be expected that proteolysis and ketogenesis would be enhanced [30]. As a result, the increment of blood concentrations of amino acids or ketone bodies is expected. Since metabolome analysis indicated that the concentrations of 3-hydroxybutyrate, a type of ketone body, between TW and BMW consumption periods were not changed, consumption of BMW may not affect generation of energy from free fatty acids. As our results demonstrated that concentrations of almost of all amino acids especially tyrosine, methionine, and glycine were decreased, but that of 3-hydroxybutyrate was not changed, BMW consumption may influence energy metabolism through proteolysis but not ketogenesis.

UDP-N-acetylglucosamine is the substrate of O-linked N-acetylglucosamine transferase and the relationship between O-linked N-acetylglucosamine transferase and insulin resistance has been previously reported [31]. However, it was also reported that concentration of UDP-N-acetylglucosamine in muscle tissue was increased after reaching euglycemia by insulin treatment in obese subjects [32]. These reports suggested that UDP-N-acetylglucosamine is related to glucose control and/or insulin resistance, but the details are still unclear. In the current study, metabolome analysis indicated that blood concentrations of UDP-N-acetylglucosamine were significantly increased after BMW consumption, but future studies are required to understand the meaning of this phenomenon.

Since recent studies reported the relationships between gut microbiota and T2D and/or obesity [33–35], we hypothesized that beneficial effect for glycemic control derived from BMW consumption might involve gut microbiota. As expected, alterations of gut microbiota compositions derived from BMW consumption were observed. In this study, family Christensenellaceae was the most significantly increased taxon. Previous study reported that Christensenellaceae was enriched in lean group (BMI < 25) as compared with obese group (BMI > 30) [36]. Additionally, it was also reported that transplantation of* Christensenella minuta* to germ-free mice reduced weight gain. Moreover, abundance of the family Dehalobacteriaceae that was also increased after BMW consumption has been reported to be positively correlated with Christensenellaceae. Therefore, our results suggested that consumption of BMW has a possible potential to prevent getting obese via increments of the abundance of lean-inducible bacteria, Christensenellaceae and Dehalobacteriaceae.

## 5. Conclusions

In this study, we have shown that serum glycoalbumin levels were significantly decreased after BMW consumption as compared with TW consumption. It was also observed that 19 blood metabolites were significantly changed and lean-inducible bacteria were significantly increased after BMW consumption (see Figure S3 of Supplementary Material). The current study is expected to become important evidence detailing the molecular level effects of BMW consumption. Finally, we believe that the molecular level elucidation of the beneficial effects derived from balneotherapy is vital for further progress and understanding of balneology and our study is the first step towards this approach.

## Supplementary Material

In the supplementary material, we showed the anthropometric characteristics of volunteers, schematic representation of the experimental design, blood amino acid concentrations and overview of the effects derived from bicarbonate-rich mineral water consumption.

## Figures and Tables

**Table 1:** Mineral contents and pH of TW and BMW used in this study.

Minerals	Formula	Conc. (mg/kg)	
		TW	BMW
Bicarbonate ion	HCO_3_^−^	28	2485
Chlorine ion	Cl^−^	11	182
Sulfate ion	SO_4_^2−^	6.9	355
Carbonate ion	CO_3_^2−^	<0.1	1.2
Nitrate ion	NO_3_^−^	0.7	1.2
Fluoride ion	F^−^	<0.1	0.3
Hydrogen sulfide ion	HS^−^	<0.1	<0.1
Iodide ion	I^−^	<0.1	<0.1
Bromide ion	Br^−^	<0.1	<0.1
Sodium ion	Na^+^	10	412
Magnesium ion	Mg^2+^	1.9	291
Calcium ion	Ca^2+^	6.1	177
Potassium ion	K^+^	<0.1	80
Aluminum ion	Al^3+^	0.2	0.6
Manganese ion	Mn^2+^	<0.1	0.4
Ferrous ion	Fe^2+^	<0.1	2.3
Ferric ion	Fe^3+^	<0.1	<0.1
Metasilicic acid	H_2_SiO_3_	10	207
Metaboric acid	HBO_2_	0.8	6.2
Carbon dioxide	CO_2_	0.9	161
Hydrogen sulfide	H_2_S	<0.1	<0.1
Mercury	Hg	<0.0005	<0.0005
Arsenic	As	<0.005	0.005
Copper	Cu	<0.05	<0.05
Zinc	Zn	<0.1	<0.1
Lead	Pb	<0.05	<0.05
Cadmium	Cd	<0.01	<0.01

pH		7.58	7.07

**Table 2:** Results of clinical blood test.

Tests	TW		BMW		*P* value
	Average	SD	Average	SD	
Fasting blood glucose (plasma) (mg/dL)	91.2	4.3	88.6	4.7	0.156
Blood glucose (serum) (mg/dL)	93.5	23.7	91.4	29.0	0.092
Glycoalbumin (% of total albumin)	14.4	1.7	14.2	1.7	0.002^*∗∗*^
Insulin (*μ*U/mL)	19.3	28.0	18.8	31.0	0.294
HOMA-R	1.2	0.5	1.2	0.4	1.000
Total cholesterol (mg/dL)	200.6	26.0	202.4	30.3	0.914
HDL cholesterol (mg/dL)	60.9	14.9	60.1	14.1	0.685
LDL cholesterol (mg/dL)	112.0	22.5	112.3	24.1	1.000
Triglycerides (mg/dL)	151.2	142.1	180.7	159.0	0.123
Urate (mg/dL)	5.2	1.2	5.2	1.2	0.396
Na (mEq/L)	139.8	1.3	140.3	1.2	0.172
Cl (mEq/L)	102.6	1.7	102.9	1.9	0.457
Ca (mg/dL)	9.3	0.2	9.4	0.2	0.021^*∗*^
Mg (mg/dL)	2.3	0.1	2.3	0.1	0.518
Cortisol (*μ*g/dL)	10.1	4.0	10.0	3.6	0.623

^*∗*^ *P* value is under 0.05; ^*∗∗*^ *P* value is under 0.005.

**Table 3:** Results of MSEA: list of metabolite sets/pathways that are significantly different between TW and BMW consumption periods.

Pathway	Total^*∗*1^	Hits^*∗*2^	Expect^*∗*3^	Fold change (hits/expect)	*P* value	FDR^*∗*4^
Purine metabolism	45	7	0.98	7.1	<0.001	0.001
Ammonia recycling	18	4	0.39	10.2	<0.001	0.014
Urea cycle	20	4	0.44	9.2	<0.001	0.014
RNA transcription	9	3	0.20	15.3	<0.001	0.014
Intracellular signaling through prostacyclin receptor and prostacyclin	6	2	0.13	15.3	0.006	0.103
Glycolysis	21	3	0.46	6.6	0.009	0.121
Citric acid cycle	23	3	0.50	6.0	0.012	0.133
Methionine metabolism	24	3	0.52	5.7	0.013	0.133
Gluconeogenesis	27	3	0.59	5.1	0.018	0.163
Amino sugar metabolism	15	2	0.33	6.1	0.040	0.290
Mitochondrial electron transport chain	15	2	0.33	6.1	0.040	0.290

^*∗*1^Total numbers of metabolites that corresponded in each pathway.

^*∗*2^Observed numbers of metabolites that derived from given dataset in each pathway.

^*∗*3^Expected observed numbers of metabolites that are calculated by given dataset in each pathway.

^*∗*4^False discovery rate (FDR) according to the Benjamini and Hochberg method that was provided by MSEA software.
