# Supplementary material for: The Consumption of Bicarbonate-Rich Mineral Water Improves Glycemic Control
Source: Evid Based Complement Alternat Med. 2015 Dec 21;2015:824395. doi: 10.1155/2015/824395 (PMC4698932; doi:10.1155/2015/824395)
Supplement: Supplementary file 1 — In the supplementary material, we showed the anthropometric characteristics of volunteers, schematic representation of the experimental design, blood amino acid concentrations and overview of the effects derived from bicarbonate-rich mineral water consumption. [file 824395.f1.pdf]

Supplementary Material

The consumption of bicarbonate-rich mineral water improves glycemic control

Shinnosuke Murakami[1,2], Yasuaki Goto[3], Kyo Ito[4], Shinya Hayasaka[3,5], Shigeo Kurihara[3], Tomoyoshi Soga[1,2], Masaru Tomita[1,2], Shinji Fukuda[1,2]

1. Systems Biology Program, Graduate School of Media and Governance, Keio University, 5322, Endo, Fujisawa, Kanagawa 252-0882, Japan
2. Institute for Advanced Biosciences, Keio University, 246-2, Mizukami, Kakuganji, Tsuruoka, Yamagata 997-0052, Japan
3. Onsen Medical Science Research Center, Japan Health and Research Institute, 1-29-4, Kakigaracho, Nihonbashi, Chuo-ku, Tokyo 103-0014, Japan
4. Ito Medical Office, 7985-5, Nagayu, Naoirimachi, Taketa, Oita 878-0402, Japan
5. Faculty of Human Life Sciences, Tokyo City University, 8-9-18, Todoroki, Setagaya-ku, Tokyo 158-8586, Japan

Correspondence should be addressed to Shinnosuke Murakami; mushin@sfc.keio.ac.jp and Shinji Fukuda; sfukuda@sfc.keio.ac.jp

Table S1. Anthropometric characteristics of volunteers

Averages and standard deviations (SD) of each parameter during 19 volunteers were calculated and shown.

	Average	SD
Age	46.9	11.1
Weight (kg)	63.0	11.8
Height (cm)	165.0	10.1
BMI (kg/m^2)	23.0	3.2
Abdominal circumference (cm)	83.8	7.5
Blood pressure (top) (mmHg)	129.7	14.9
Blood pressure (bottom) (mmHg)	76.1	8.6

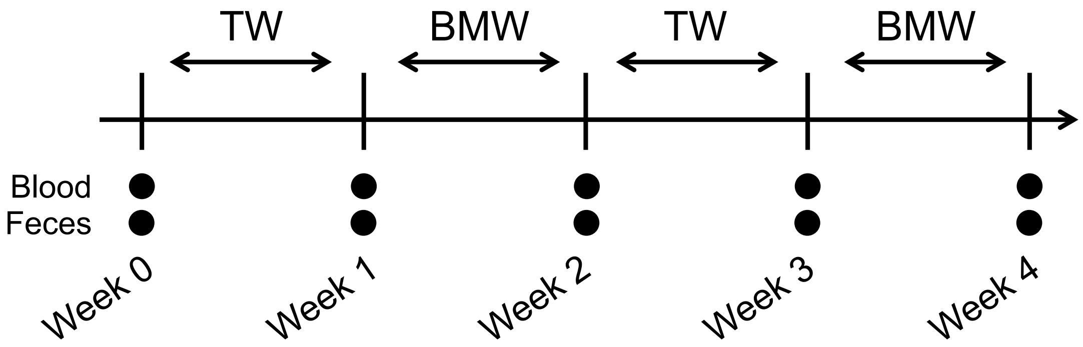

Figure S1. Schematic representation of the experimental design.

BMW consumption test was taken 4 weeks. TW consumption periods and BMW consumption periods lasted for a week each and this cycle was repeated twice. Blood and fecal samples were collected on the first day of the test and last days of every week.

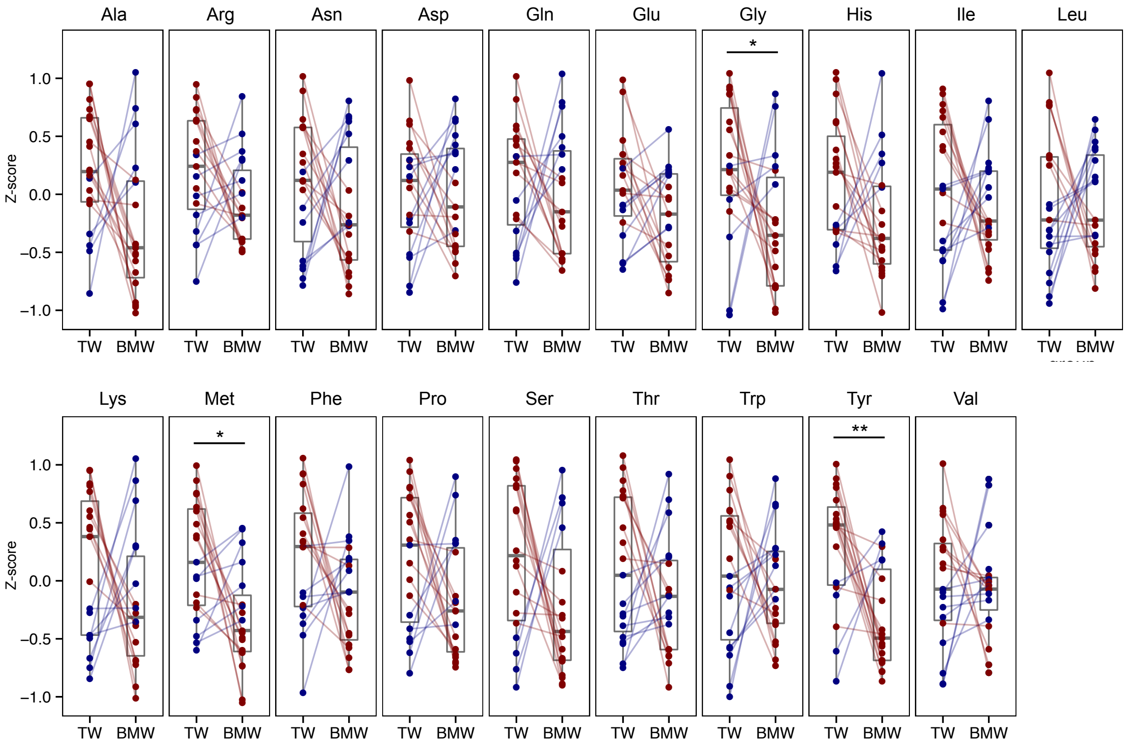

Figure S2. Comparisons of blood amino acids between TW and BMW consumption periods.

Mean relative concentrations of each amino acid (Z-score) of week 1 and 3 (TW) and week 2 and 4 (BMW) were shown in dot plots overlaid on box plots. Plots corresponding to the same individuals were connected with red, blue or gray lines when the values were decreased, increased or not changed in BMW consumption periods as compared with TW consumption periods, respectively. Plots were also colored in the same color as their lines. Relative concentrations of standard amino acids aside from cystein were shown because cystein was not detected by CE-TOFMS measurement. *P<0.05; **P<0.005.

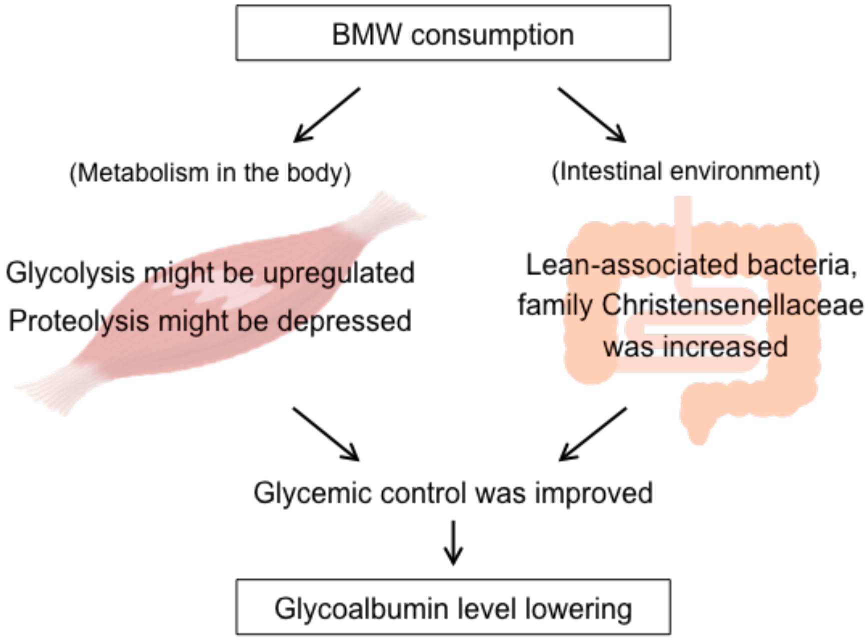

Figure S3. Overview of the effects derived from BMW consumption.

We speculated the mechanisms how BMW consumption lead reduction of serum glycoalbumin levels. According to the results of metabolome analysis, glycolysis was upregulated. Lowering of blood amino acids may be attributed to depression of proteolysis. Additionally, lean-inducible bacteria such as family Christensenellaceae were increased after consumption of BMW. Therefore, combination of these effects might improve glycemic control and thereby result in reduced serum glycoalbumin levels.
